# A Survey on Probabilistic Models in Human Perception and Machines

**DOI:** 10.3389/frobt.2020.00085

**Published:** 2020-07-07

**Authors:** Lux Li, Robert Rehr, Patrick Bruns, Timo Gerkmann, Brigitte Röder

**Affiliations:** ^1^Biological Psychology and Neuropsychology, University of Hamburg, Hamburg, Germany; ^2^Signal Processing (SP), Department of Informatics, University of Hamburg, Hamburg, Germany

**Keywords:** signal processing, multisensory perception, audiovisual integration, optimal cue integration, causal inference, speech enhancement, automatic speech recognition, human psychophysics

## Abstract

Extracting information from noisy signals is of fundamental importance for both biological and artificial perceptual systems. To provide tractable solutions to this challenge, the fields of human perception and machine signal processing (SP) have developed powerful computational models, including Bayesian probabilistic models. However, little true integration between these fields exists in their applications of the probabilistic models for solving analogous problems, such as noise reduction, signal enhancement, and source separation. In this mini review, we briefly introduce and compare selective applications of probabilistic models in machine SP and human psychophysics. We focus on audio and audio-visual processing, using examples of speech enhancement, automatic speech recognition, audio-visual cue integration, source separation, and causal inference to illustrate the basic principles of the probabilistic approach. Our goal is to identify commonalities between probabilistic models addressing brain processes and those aiming at building intelligent machines. These commonalities could constitute the closest points for interdisciplinary convergence.

## Introduction

Human perception and machine signal processing (SP) both face the fundamental challenge of handling uncertainty. Probabilistic models provide powerful tools for representing and resolving uncertainty (Rao et al., 2002). For example, a simple probabilistic model for estimating a speech signal from a noisy audio recording can be constructed as follows: The stimulus parameter of interest (e.g., the phoneme) is represented as a latent variable *S*. The existing information or expectation regarding *S* prior to the data observation is represented by the prior probability distribution, *p(S)* (“*prior*”). The perceptual system's responses (often referred to as *measurements*) are usually stochastic: they fluctuate from trial to trial even when the stimulus remains constant. The conditional probability density function (PDF) of obtaining the measurements *X* given *S* is described by the likelihood function of *S, p(X| S)* (“*likelihood*”). Probabilistic models commonly use the framework of *Bayesian inference*, which specifies how belief is optimally updated in light of new evidence. Computationally, this is achieved by applying the Bayes' theorem (Pouget et al., 2013; Ghahramani, 2015) to combine the likelihood and the prior to calculate the posterior probability distribution (“*posterior*”), *p(S |X)*:

(1) $$\begin{array}{l} \text{p}\left(\text{S|X}\right)=\text{p(X|S) p}\left(\text{S}\right)/\text{p}\left(\text{X}\right) \end{array}$$

Signal reconstruction often requires a point-estimator for *S*. Three methods are commonly used. The maximum likelihood estimator (MLE) is the *S* value that maximizes the likelihood (Equation 2) or equivalently the log-likelihood, implying a uniform (flat) prior. The maximum *a-posteriori* (MAP) estimator can be seen as maximizing the likelihood after factoring in an informative prior (Equation 3) and is equal to the posterior mode. The minimum mean square error (MMSE) estimator is the *a-posteriori* expected value for *S* (Equation 4) and is equal to the posterior mean (Yuille and Bülthoff, 1996; Maloney, 2002).

(2) $$\begin{array}{l} \text{MLE}:Ŝ=arg\;\underset{s_{i}}{\max}\;p\left(X|S_{i}\right) \end{array}$$

(3) $$\begin{array}{l} \text{MAP}:Ŝ=arg\;\underset{s_{i}}{\max}\;p\left(S_{i}|X\right) \end{array}$$

(4) $$\begin{array}{l} \text{MMSE}:Ŝ=\int S_{i}\;p\left(S_{i}|X\right)dS_{i} \end{array}$$

Similar probabilistic approaches are applied in sensory perception and machine SP for solving analogous problems, such as robust perception. However, although recent reviews have separately summarized probabilistic models in each of these disciplines (Kolossa and Häb-Umbach, 2011; Ma, 2012; Hendriks et al., 2013; Ursino et al., 2014), reviews that draw parallels between the models across the disciplines are lacking. Here, we will introduce and compare selective applications of probabilistic models in psychology, neuroscience, and machine SP, focusing on audio and audio-visual processing. We use the topics of speech enhancement, automatic speech recognition, audio-visual cue integration, and source separation as examples, because probabilistic models have played a particularly important role in advancing these research areas. We emphasize two important aspects of resolving uncertainty: noise reduction and source separation. While in recent years machine learning approaches have had a great impact in SP (Deng and Li, 2013; Padmanabhan and Premkumar, 2015), neuroscience (Yamins and DiCarlo, 2016), and cognitive science (Lake et al., 2017), here we highlight the commonalities between basic probabilistic models for machine and perceptual SP.

## Noise Reduction and Speech Enhancement

Statistical approaches in speech enhancement for reducing background noise usually deal with single-channel signals, e.g., from a single microphone. The variance of a signal is generally understood as the power of the signal, and the PDFs characterize the coefficients of the digitized signals. Traditionally, the complex Fourier coefficients of the speech and noise components are modeled with a zero-mean Gaussian distribution [but later research suggests that super-Gaussian PDFs are more appropriate; see Lotter and Vary (2005); Martin (2005), and (Rehr and Gerkmann, 2018)], and the frequency bands are assumed to be statistically independent (Ephraim and Malah, 1984, 1985; Porter and Boll, 1984). The variances (i.e., the power) of the speech and noise coefficients are time-variant; therefore, the parameters must be continuously updated using adaptive power estimators. A common way to derive the estimators is by computing the MMSE between the true speech coefficients and the estimated coefficients, which leads to a linear filter known as the Wiener filter (Ephraim and Malah, 1984; Martin, 2001; Gerkmann and Hendriks, 2012). The Wiener filter has been adapted for multi-channel (e.g., multi-microphone array) processing (Krawczyk-Becker and Gerkmann, 2016), which additionally allows exploiting the spatial properties of sound (Kay, 1993; Balan and Rosca, 2002; Doclo et al., 2015). For multi-channel noise reduction, a well-known concept is the minimum-variance distortionless response (MVDR) beamformer. This beamformer minimizes the power of the output signal while ensuring that the sounds from the target speaker are not distorted or suppressed. The MVDR beamformer can be derived as the MLE of the speech coefficients if the background noise is assumed to follow a multivariate complex Gaussian distribution (Kay, 1993; Balan and Rosca, 2002).

Another classical probabilistic approach for estimating speech and noise coefficients is to use mixture models, most commonly Gaussian mixture models (GMMs) and hidden Markov models (HMMs) (Rabiner, 1989), with machine-learning methods (Ephraim, 1992; Burshtein and Gannot, 2002; Zhao and Kleijn, 2007; Chazan et al., 2016). The time-varying speech components are characterized by a sequence of discrete states related to the phonemes uttered by a speaker. Each state is described by a PDF linking it to the statistics of the observations. GMMs explicitly quantify the joint contributions of different states, whereas HMMs treat the states as latent variables that are related through Markov processes. The resulting estimator is a mixture of clean speech estimates from all possible combinations of available states; the states that best explain the observations have the strongest influence on the overall estimate. The advantage of a mixture estimator is that it takes into account all possible states and is more robust than basic MLEs.

Auditory systems of animals maintain robust neuronal representation of relevant sounds in noisy environments (Mesgarani et al., 2014). The dominant model for characterizing auditory neuronal responses is the spectrotemporal receptive field (STRF) (Zhao and Zhaoping, 2011; David, 2018; King et al., 2018). STRF is a linear filter that approximates the neuronal response at a given time as a linear weighted sum of the stimulus power at recent time points in different spectral channels (King et al., 2018). The weights can be viewed as a discrete-time version of the Wiener filter if they are estimated via the MMSE between the model output and the measured neuronal response, assuming Gaussian response noise with constant variance (Meyer et al., 2017). STRF is usually applied as part of a linear-nonlinear (LN) model—linear input followed by static nonlinear response generation (Chichilnisky, 2001; Paninski, 2003; Sharpee et al., 2004). However, standard STRF and LN models do not incorporate the highly nonlinear and dynamic neural processes which are important for noise robustness (for reviews, see Meyer et al., 2017; King et al., 2018). For example, auditory neurons adapt to stimulus statistics, such as the mean level and the contrast (i.e., the sound level variance) of recent sounds, and adjust their sensitivity accordingly; this adaptation enables efficient and robust neural coding (Fritz et al., 2003; David et al., 2012; Rabinowitz et al., 2013; Willmore et al., 2014, 2016; Lohse et al., 2020). STRF models extended with adaptive kernels (Rabinowitz et al., 2012) and other nonlinear features, such as input nonlinearity (Ahrens et al., 2008), synaptic depression (Mesgarani et al., 2014), gain normalization (Mesgarani et al., 2014), or top-down influence, such as feedback (Calabrese et al., 2011) and selective attention (Mesgarani and Chang, 2012), have been shown to better account for noise robustness. In addition, mixture-model approaches from SP (e.g., GMM) can be used to scale these models to higher-dimensional stimuli (Theis et al., 2013). In machine SP, machine-learning algorithms inspired by the nonlinear, adaptive, and/or top-down features of auditory neurons are being developed to improve speech enhancement (Ephraim, 1992; Hendriks et al., 2013; Lee and Theunissen, 2015; Rehr and Gerkmann, 2018, 2019). Future research could aim at building brain-inspired robust and flexible models to cope with various noise types, cluttered real-world data, and adversarial data.

## Audio-Visual Integration Models in a Single-Source Setup

Probabilistic approaches have been extensively used for automatic speech recognition (ASR): the translation of audio signals into written text. Identifying the spoken words based only on the acoustic input signal can be challenging, especially if noise is present. Incorporating visual information (e.g., mouth shape, lip movement) can substantially improve ASR performance (Hennecke et al., 1996) in noisy environments, because visual features provide contextual and complementary (but additionally redundant) information about the audio scene and are insensitive to the acoustic background noise (Nefian et al., 2002). This approach is known as audio-visual speech recognition (AVSR). AVSR systems require dynamic models for optimal audio-visual (AV) integration. The performance of conventional HMMs, although being time-flexible, is limited by their strong restrictive assumptions, e.g., that the signal-generating system is a single process with few states and an extremely limited state memory (Brand et al., 1997). Nevertheless, a variety of HMM extensions have been proposed to better solve the AV fusion problem (Potamianos et al., 2003). One approach is to use a combination of feature fusion and decision fusion (Neti et al., 2000; Potamianos et al., 2003). *Feature fusion* applies fusion on the feature level; it trains a single HMM classifier on the concatenated vector of audio and visual features (Adjoudani and Benoît, 1996). *Decision fusion* applies fusion on the classifier output level; it linearly combines the likelihoods of audio-only and visual-only streams into a joint AV likelihood, using weights that capture the reliability of each sensory modality (Jain et al., 2000; Neti et al., 2000). Measures of reliability include the inverse variance (Hershey et al., 2004), signal-to-noise ratio (Adjoudani and Benoît, 1996; Hennecke et al., 1996), harmonics-to-noise ratio (Yumoto et al., 1982), or an equivalent index (Neti et al., 2000).

Two other extensions of HMMs are coupled HMMs (Brand et al., 1997; Abdelaziz et al., 2015) and factorial HMMs (Ghahramani and Jordan, 1997). These models have several advantages over conventional HMMs for AVSR: (1) they allow state asynchrony between the audio and visual components while preserving their natural correlation over time (Nefian et al., 2002; Abdelaziz et al., 2015), (2) they can model multiple interacting processes without violating the Markov condition (Brand et al., 1997), (3) the distributed state representations employed by these models allow automatic decomposition of superposed states (Ghahramani and Jordan, 1997), and (4) they are less sensitive to the initial conditions of parameters (Brand et al., 1997).

**Figure 1:**
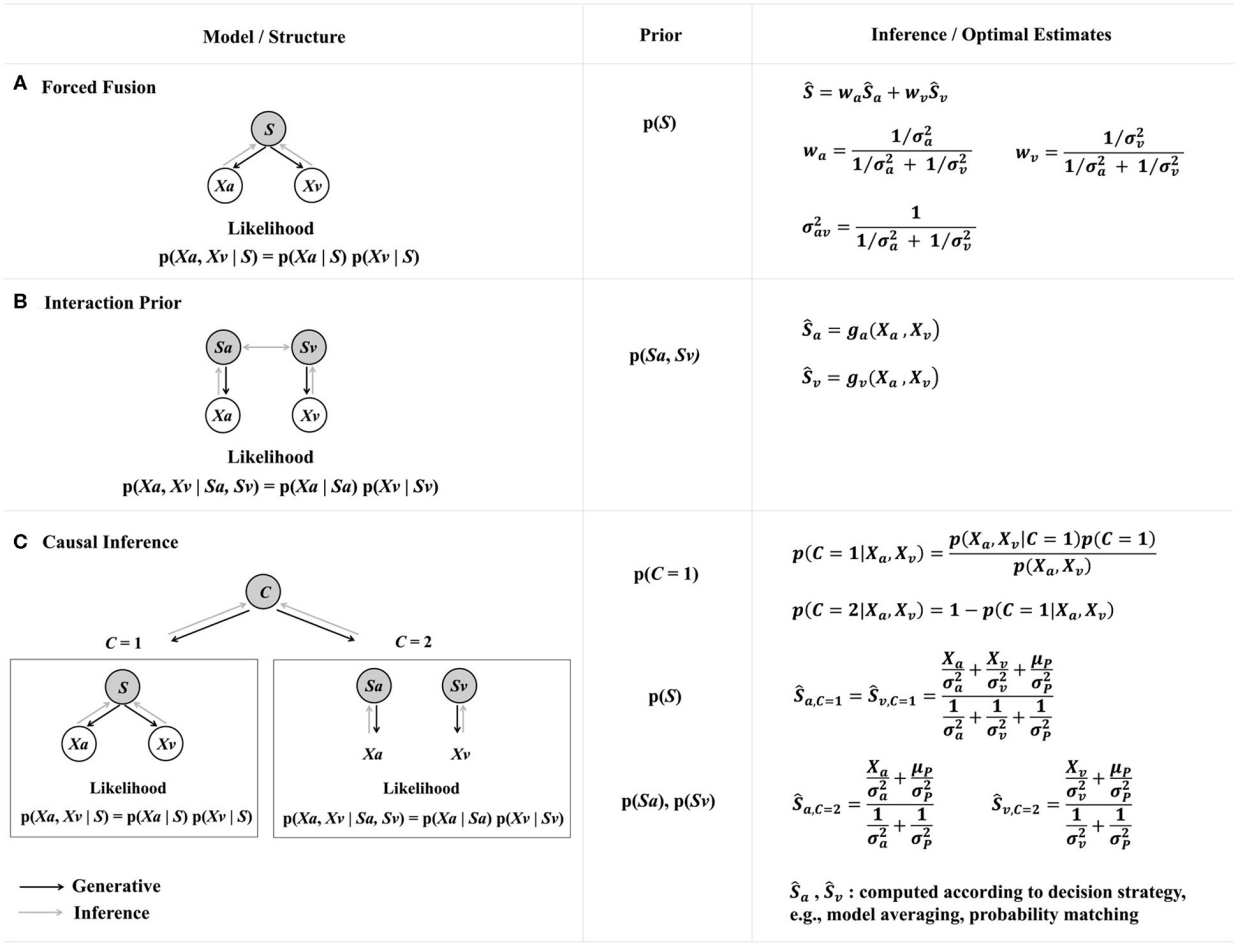
Three probabilistic models for audio-visual cue integration in human psychophysics. Gray nodes depict the latent stimulus attribute *S* (e.g., identity or position) or the latent causal structure *C*. White notes depict the sensory measurements *X* in response to the sensory cues (*a*: auditory, *v*: visual). Left panel: The generative models and the underlying structures. The likelihood functions are derived under the assumptions that the auditory and visual cues are corrupted by independent Gaussian noise. Black arrows represent the direction of generative process, and gray arrows represent the direction of inference. Middle panel: A-priori knowledge. Right panel: Optimal estimates by Bayesian inference (adapted from Ursino et al., 2014 Box 1, copyright © 2014 Elsevier Ltd, and Shams and Beierholm, 2010 Figure 1, copyright © 2010 Elsevier Ltd; reused with permission). **(A)** Forced fusion model. The auditory and visual cues are assumed to have a common cause. The prior is usually assumed to be uniform, in which case this model is equivalent to an MLE. The optimal estimate is a linear weighted summation of unimodal MLEs, and the weights are the relative cue reliabilities (precision). This model describes complete cue integration (fusion). **(B)** Interaction prior model. The joint prior distribution *p*(*S*_*a*_*, S*_*v*_) reflects the prior knowledge about the audio-visual correspondence in the environment. A common choice is a 2D Gaussian or Gaussian-mixture function with higher probabilities along the identity line *S*_*a*_ = *S*_*v*_. The estimates could be linear or non-linear functions (*g*_*a*_, *g*_*v*_) depending on the specific interaction prior. This model can describe complete fusion, partial integration, or segregation of cues. **(C)** Causal inference model. The latent variable *C* determines the causal structure that generates the cues and mediates cue integration: cues are integrated if they have a common cause (*C* = 1) and processed separately if they have independent causes (*C* = 2). The model infers the probability of the unknown causal structure p(*C* |*X*_*v*_, *X*_*a*_) and weights the estimates Ŝ_*a*_ and Ŝ_*v*_ accordingly using some decision strategy (Wozny et al., 2010). The estimates are nonlinear combinations of the cues and usually require Monte Carlo simulation to obtain (Körding et al., 2007). This model can be recast as the coupling prior model **(B)** by integrating out the latent variable *C*, in which case it will no longer explicitly represent the causal structure.

AVSR models are inspired by the human ability of using visual information to reduce auditory ambiguity (Schwartz et al., 2004). In human perception, a research topic related to AV fusion is generally known as *cue integration*. A *cue* is a sensory signal that bears information about the state of some stimulus property, e.g., identity or position. Psychophysical and neurophysiological studies have shown that the brain combines multiple cues both within and across sensory modalities to reduce uncertainty (for a review, see Fetsch et al., 2013). Computationally, to reduce uncertainty means to minimize the variance of perceptual estimates. One of the most well-known computational models for cue integration in psychophysics is the *forced fusion model* (Figure 1A), also known as the optimal cue integration model or the MLE model. This model proposes that a minimum-variance estimate for the target stimulus attribute *S* given multiple cues can be computed as the weighted linear sum of the MLEs for individual cues, and the weights are determined by each cue's relative reliability (Alais and Burr, 2004; Ernst and Bülthof, 2004; Rohde et al., 2015). A cue's *reliability* is defined as its inverse variance, $\frac{1}{\sigma_{i}^{2}}$, which is akin to how reliability is defined in a MVDR beamformer (Kay, 1993; Balan and Rosca, 2002). The forced fusion model assumes that the cues are *redundant*, i.e., they are regarding a single stimulus attribute and therefore should be completely integrated. Under the simplifying assumptions of a uniform prior *p*(*S*) and independent Gaussian noises, the posterior *p*(*S* | *X*_1_, *X*_2_, …, *X*_n_) is also a Gaussian, with its mean given by weighted summation:

(5) $$\begin{array}{l} {Ŝ}_{opt}=\overset{n}{\underset{i=1}{\sum }}{w_{i}Ŝ}_{i}\;,w_{i}=\frac{\frac{1}{\sigma_{i}^{2}}}{\overset{n}{\underset{i}{\sum }}\frac{1}{\sigma_{i}^{2}}} \end{array}$$

where Ŝ_*opt*_ is the optimal combined estimate, Ŝ_*i*_ is the MLE for an individual cue *i*, and *w*_*i*_ is the weight determined by the relative reliability of cue *i*. These weights (*w*_*i*_) minimize the variance of the combined estimate, and thus Ŝ_*opt*_ is a minimum-variance unbiased estimator for *S* [for a mathematical proof, see Colonius and Diederich (2018)]. This forced fusion model is analogous to the aforementioned fusion models used in multi-stream HMM for AVSR (Neti et al., 2000). The reliability-based weighting is similar to the stream weights that are determined by the inverse variance (Hershey et al., 2004). However, in the forced fusion model the weights are fixed, while in AVSR it has been shown that dynamic stream weights resulted in better performance (Meutzner et al., 2017)_._ Furthermore, even in the seemingly simple case of fusing information from multiple microphones, the noise captured by individual microphones is typically correlated, especially in low frequencies. As a consequence, the minimum-variance estimate typically takes into account the full correlation matrices of the noise (Doclo et al., 2015).

Recent psychophysical research has suggested that the MLE-type complete fusion is not a general property of human multisensory perception (e.g., Battaglia et al., 2003; Arnold et al., 2019; Meijer et al., 2019). To capture the full spectrum of cue interaction spanning from complete fusion to partial integration to segregation, extensions of the forced fusion model have been proposed. Among them, the *coupling prior model* (Figure 1B), also known as the interaction prior model, extends the forced fusion model (Figure 1A) by adding a joint prior distribution to represent the correlation or co-occurrence statistics between the cues (Shams et al., 2005; Rowland et al., 2007; Ernst, 2012; Parise et al., 2014). For example, in a speech recognition task with auditory and visual cues, a coupling prior model could use a bivariate prior *p(S*_*a*_*, S*_*v*_*)* to describe the joint probability distribution for the auditory (*S*_*a*_) and visual (*S*_*v*_) representations of the stimulus attribute (e.g., syllables). The coupling prior can be conveniently modeled using a 2D Gaussian *p(S*_*a*_*, S*_*v*_*)* = $N_{Sa,\;Sv}\left(\bar{s},\;\Sigma \right)$, with the mean $\bar{s}$ being the expected stimulus value, and the covariance matrix Σ consisting of variances along the principle axes (e.g., Ernst, 2007). The *p(S*_*a*_*, S*_*v*_*)* distribution is sharper if the AV coupling is relatively constant (due to statistical regularities in the environment or acquired through adaptation or learning). The forced fusion model is a special case of the coupling prior model where *p(S*_*a*_*, S*_*v*_*)* = 0 for all *S*_*a*_ ≠ *S*_*v*_. Another method for characterizing the coupling prior is to use a GMM to represent the correlated and the uncorrelated components (e.g., Roach et al., 2006; Sato et al., 2007); the resulting mixture estimator is more general and robust than MLE.

The coupling prior model for cue integration is analogous to a GMM for AVSR, where the AV coherence (i.e., dependency between the auditory and visual modalities) is expressed as a joint AV PDF (Rivet et al., 2014). It can be viewed as loosely similar to the basic concept of coupled HMMs for AVSR, too. However, unlike coupled HMMs, the coupling prior model is not dynamic and does not describe time-variant signals. Moreover, the coupling prior model explicitly constrains the joint prior distribution of the cues, whereas coupled HMMs implicitly learn the hidden states that generate the cues.

## Source Separation and Causal Inference

In machine SP, the most common scenario of source separation is *blind source separation* (BSS): separating two or more source signals given mixture observations (Jutten and Herault, 1991; Castella et al., 2010). A fundamental challenge in BSS is the *label permutation problem*: to track which speech signal belongs to which speaker/source (Hershey et al., 2016). To achieve this, a model needs to jointly solve two problems: isolating a single speech signal from a dynamic mixture of sounds from multiple speakers and the background noise, and assigning the speech signal to the corresponding speaker (Ephrat et al., 2018). A Bayesian approach to solve BSS is applying GMMs and HMMs that either constrain or learn the unobservable source structure underlying the mixture signals (Roweis, 2001, 2003; Hershey and Casey, 2002; Yilmaz and Rickard, 2004). Inspired by human perception, recent machine SP models have been exploiting the intrinsic AV coherence to improve BSS performance (Rivet et al., 2014). Full joint AV models based on maximizing the AV likelihood can successfully extract source signals from underdetermined mixtures (Sodoyer et al., 2002). However, such models are limited to *instantaneous* mixtures, where multiple source signals contribute to the mixtures without delay at a given time point. Similarly in human perception, most existing mixture models for cue integration consider only instantaneous mixtures (e.g., Magnotti and Beauchamp, 2017). If multiple source signals contribute to the mixtures with different levels of delay—known as *convolutive* mixtures—alternative techniques are required to resolve the added ambiguities in BSS (e.g., Rivet et al., 2007; Liu et al., 2012. For a review, see Rivet et al., 2014).

In natural environments, the structure of the source(s) giving rise to the signals is often ambiguous or unobservable; therefore, to properly associate a signal with its source, the observer needs to infer cause-effect relationships based on the noisy data. This is an example of the so-called *inverse problem* in information processing: inferring the cause given the effect (Ghahramani, 2015). Humans are remarkably apt at solving this problem, being able to focus on a target speaker while filtering out interfering sounds and background noise, as exemplified by the well-known cocktail party effect (Cherry, 1953). However, the causal inference problem is challenging for machine SP, especially in AVSR, as it is difficult to determine which signals in the mixture data came from the same source and thus should be fused.

Machine SP could draw inspiration from the *causal inference model* in human psychophysics (Figure 1C), which explicitly characterizes the hidden causal structure of the source signal(s) (Körding et al., 2007; Shams and Beierholm, 2010; Magnotti and Beauchamp, 2017). This model proposes that humans estimate the hidden causal structure based on statistical regularities of the environment and use this estimate to arbitrate between grouping or segregating sensory cues (Noppeney and Lee, 2018). The basic structure of this model has two hierarchies. In the higher hierarchy is a binary latent variable representing whether the multiple cues share a common cause, denoted as *C* (short for “cause”). *C* = 1 means the cues have a common cause, and *C* = 2 means the cues have two separate causes. The *a-priori* belief for *C* is the *causal prior*, and it influences whether and to which degree cues are integrated: cues are integrated only if they have a common cause, in which case the model is equivalent to a forced-fusion MLE model (Figure 1A); in contrast, the cues are processed separately if they originate from different causes. The causal structure is unknown, so the model needs to infer *C* by combining bottom-up sensory data with top-town causal priors and calculating the posterior *p*(*C*|*X*_*a*_, *X*_*v*_) for different *C* values. The model additionally computes the PDF for the task-relevant estimate *p*(Ŝ|*X*_*a*_, *X*_*v*_, *C*) under the assumption of common or separate causes, respectively. A final estimate for the stimulus attribute is obtained by combining these estimates according to some decision strategy. For example, if a model-averaging decision strategy is applied, which is based on the use of MMSE, then the resulting final estimate is the weighted average of the estimates obtained under *C* = 1 and *C* = 2, respectively, with the weights being the corresponding posterior probabilities for *C* = 1 and *C* = 2 (Körding et al., 2007; Wozny et al., 2010).

## Summary and Outlook

Here we reviewed a selection of probabilistic models of audio- and AV-processing applied in machine SP and in human perception, focusing on speech enhancement, speech recognition, cue integration, and causal inference (Table 1). In their cores, these models are stimulus-response functions: they describe a probability distribution of responses given a stimulus and parameters, and the parameters can be estimated from experimental data or machine learning methods. Basic probabilistic models are often linear filters with Gaussian PDFs (e.g., Wiener filter, classic STRF), which can be extended with nonlinear, adaptive, and/or top-down features (e.g., super-Gaussian prior, gain control, selective attention). In addition, the use of mixture models (e.g., GMM, HMM) simultaneously accounts for multiple possible states and permits more robust parameter estimation. Furthermore, basic probabilistic models can be adapted to characterize multiple input channels or streams (e.g., MVDR beamformer). If multiple inputs are combined (e.g., cue integration, AVSR), fusion models with reliability-based weighting and MLE are typically applied. However, forced fusion is not always appropriate. Therefore, to capture the large spectrum of input interactions, some models incorporate the correlation between the inputs (e.g., coupling prior model, coupled or factorial HMM) instead of assuming fusion. Moreover, causal inference models estimate the hidden source or causal structure of the inputs, by factoring in causality which is important for determining input integration or source separation. More advanced models, such as those in machine learning, are beyond the scope of this mini review. In short, this brief tutorial linked the analogous counterparts among probabilistic models developed in artificial and natural systems and identified the closest points of potential overlap between these models.

**Table 1:** An overview of selective probabilistic models of audio- and audio-visual (AV) processing in machines and human perception.

	**Problem**	**Model**	**Main features and advantages**	**Limitations**
**Noise reduction and speech enhancement**				
Machine speech enhancement	Estimation of speech coefficients	Wiener filter with simple Gaussian PDFs	Linear, low computational cost, easy to implement	Gaussian PDFs not appropriate for modeling speech Fourier coefficients. Super-Gaussian is better
		MVDR beamformer	Suitable for multi-channel noise reduction	
		GMM	Dynamics of speech and noise captured by states of a mixture model. Mixture estimator	Typically restricted to a small number of classes; limited robustness in reverberant conditions
		HMM	Improves modeling of temporal behavior by including state transitions. Mixture estimator	Strong restrictive assumptions, intolerant to state asynchrony in AV combined streams, sensitive to initial parameter values
Auditory neural processing	Maintaining robust neuronal representation of relevant sounds	Spectrotemporal receptive field (STRF)	Computational simplicity, analytic tractability, interpretability	Does not capture the highly nonlinear and dynamic features of auditory neurons
**Audio-visual (AV) integration and speech recognition**				
Machine ASR	ASR	GMM/HMM	Captures the dynamics of speech	Other modalities cannot be easily included
	AVSR	Coupled HMM, factorial HMM	Improves AV fusion over conventional HMM for AVSR	
Human AV integration	Optimal AV cue combination	Forced fusion (MLE) model	Reliability-weighting, minimum-variance unbiased estimator	Complete fusion only; does not account for cue coherence or causal structure
	Accounting for AV correlation	Coupling prior model, can use GMM	Joint AV prior distribution. Can capture the full range of AV integration	Cannot infer causal relationships
**Source separation and causal inference**				
Machine source separation	Source separation, label permutation problem	Blind source separation techniques with GMM, HMM, etc.	Does not need a-priori information about causal structure; works for instantaneous mixtures and convolutive mixtures	
Human AV integration	Causal inference	Causal inference model	Explicitly represents the underlying causal structure; more general than forced-fusion and coupling prior models	Can be computationally expensive

## Author Contributions

All authors: conceptualization, review and editing, and approval of final version. LL and RR: literature research and analysis and manuscript drafting.

## Conflict of Interest

The authors declare that the research was conducted in the absence of any commercial or financial relationships that could be construed as a potential conflict of interest.
